# Deep learning-based estimation of Kent mango yield by commercial export category under real field conditions in northern Peru

**DOI:** 10.3389/fpls.2026.1943711

**Published:** 2026-10-13

**Authors:** Victor Yoel Huaman Asencio, Heber Iván Mejía Cabrera, Juan Carlos Arcila Díaz

**Affiliations:** Escuela Profesional de Ingeniería de Sistemas, Facultad de Ingeniería, Arquitectura y Urbanismo, Universidad Señor de Sipán, Lambayeque, Peru

**Keywords:** computer vision, convolutional attention, export category classification, Kent mango, object detection, precision agriculture, yield estimation, YOLO

## Abstract

Manual visual counting remains the standard procedure for estimating Kent mango (*Mangifera indica* L.) yield in northern Peru, an inconsistent practice that compromises harvest planning, transport dimensioning and commercial negotiation. Existing computer vision approaches to mango detection resolve fruit-versus-background discrimination but rarely classify fruit into the commercial categories that determine its market value. This study developed and validated a deep learning method that simultaneously detects and classifies Kent mango fruit into three export categories (air freight, sea freight and reject) from RGB images acquired under real field conditions in the Motupe district, Lambayeque. A purpose-built dataset of 400 photographs was compiled from 200 trees across ten commercial farms using two opposite views per tree; 6,544 patches of 1,280 × 1,280 pixels with 20% overlap were generated, containing 66,465 manually annotated instances. Five architectures were trained under identical experimental conditions: YOLOv8m, YOLO11m, YOLO26m, RT-DETR-l and Mango-YOLO, the latter a proposed architecture built on YOLO26m through the incorporation of a CBAM attention module, a BiFPN neck and the MPDIoU regression loss. Mango-YOLO achieved the best performance across all five metrics, with a precision of 0.930, a recall of 0.940, an F1-score of 0.930, a mAP@0.5 of 0.940 and a mAP@0.5:0.95 of 0.730, at an inference time of 114.94 ms per image. Per-category analysis revealed heterogeneous behaviour, with F1-scores of 0.960, 0.845 and 0.792 for sea freight, air freight and reject respectively. The selected model was integrated into a three-tier web application that scales fruit counts from sampled trees to the whole farm and converts them into kilograms through Monte Carlo simulation with category mean weights, returning a 90% interval. Validation against harvested production recorded on the processing-plant scale across the ten farms yielded a mean absolute percentage error of 2.63% and a coefficient of determination of 0.9993, outperforming the field supervisor’s manual estimate by a factor of 3.3.

## 1 Introduction

Mango is one of Peru’s principal agro-export commodities and sustains the economy of thousands of families in the north of the country. The 2024/2025 season confirmed this with record figures of 289,508 metric tonnes of fresh mango exported, of which the Kent cultivar accounted for the overwhelming majority, with the United States, the Netherlands and Spain as leading destinations (MIDAGRI, 2025).

Lambayeque has consolidated its position as the country’s second mango-producing region, behind Piura, and within Lambayeque the Motupe district ranks second in production after Olmos (INEI, 2025). This production volume has a direct consequence for local growers, who depend on approximate estimates to plan their harvest and negotiate sales. The usual method consists of walking the plots, sampling a subset of trees and counting fruit by hand to project the total. When fruit load is high and the environment is complicated by changing light or shade, this visual count becomes inconsistent and unreliable, which in turn affects harvest planning, generates poorly dimensioned transport, improvised hiring of day labourers and unfavourable negotiations with buyers.

Computer vision and deep learning techniques offer an alternative to the manual method. Image processing has been applied in agriculture to measure canopy, estimate yield and classify produce with greater accuracy and in less time than traditional methods (Vibhute and Bodhe, 2012). Training deep models from scratch, however, requires large volumes of labelled data, a condition that is not always feasible in specific agricultural settings. Transfer learning allows models pretrained on large generic databases to be reused and adjusted to a particular task with fewer examples, accelerating convergence and improving generalization when the local dataset is limited (Pan and Yang, 2010). Complementarily, data augmentation artificially widens the variability of the training set through geometric and photometric transformations, reducing overfitting and improving performance under illumination, scale and orientation conditions different from those initially observed (Shorten and Khoshgoftaar, 2019).

In the field of object detection, the YOLO (You Only Look Once) family has evolved towards increasingly efficient architectures. YOLO11 improves feature extraction through C3k2, SPPF and C2PSA blocks, and has been applied successfully to citrus detection (Liao et al., 2025). The most recent version, YOLO26, moves towards non-maximum-suppression-free inference, eliminates the Distribution Focal Loss and proposes label assignment strategies aimed at small objects (Jocher et al., 2026). RT-DETR, in turn, combines the Transformer architecture with an efficient hybrid encoder, removing the need for non-maximum suppression and offering flexible inference speed (Zhao et al., 2024). In the specific context of mango, Light-YOLO demonstrates that lightweight and efficient models can be developed for fruit detection in natural environments (Zhong et al., 2024).

These models can be enhanced with attention mechanisms and specialized loss functions. The Convolutional Block Attention Module (CBAM) strengthens spatial and channel feature extraction, improving object discrimination in complex scenes (Woo et al., 2018). The weighted bidirectional feature pyramid network BiFPN, originally proposed in EfficientDet, facilitates efficient multi-scale feature fusion, which is advantageous for detecting objects of variable size within the same image (Tan et al., 2020). Likewise, the MPDIoU (Minimum Point Distance Intersection over Union) loss function incorporates the distance between the extreme points of the bounding boxes, improving coordinate regression when objects overlap or are small (Ma and Xu, 2023).

A review of specific antecedents allows the potential and the limitations of these techniques applied to mango to be established. A comparative analysis between YOLOv8 and Faster R-CNN for mango detection trained both models with a dataset of 212 original images expanded to 2,449 images and 116,654 annotations through data augmentation: YOLOv8 reached 96.72% precision and 77.4% recall, whereas Faster R-CNN achieved 98.57% precision but only 63.80% recall, evidencing that YOLO is faster and more balanced for this task (Arcila-Díaz et al., 2024). Recognition accuracy has also been raised through convolutional neural networks for mango yield estimation, evaluating eight crops with 800 images and reaching 98.85% accuracy on the test set (Neethi and Raviraj, 2025). In India, Faster R-CNN with a ResNet-50 backbone reached 98.20% precision for on-tree mango yield estimation, although with a small dataset that limits generalization (Vasanth and Pandian, 2024).

In Australia the problem was addressed from a more realistic perspective, focusing on the inevitable underestimation of computer vision methods. Each tree was captured from multiple angles and four estimation methods were compared: human counting of 5% of trees, counting of 18 trees selected by NDVI, uncorrected multi-view counts and multi-view counts corrected for hidden fruit, with mean percentage errors relative to packing-house counts of 26%, 13%, 11% and 17% respectively (Anderson et al., 2021). These results indicate that detecting visible fruit constitutes only part of the problem, and that correcting the number of detections for occluded fruit is indispensable if actual load is to be estimated (Sarron et al., 2023). Hyperspectral imaging constitutes another technological avenue: in an Australian orchard a ground vehicle equipped with an HSI sensor produced models validated on hundreds of trees that reached coefficients of determination of up to 0.75 against manual counts and 0.83 against standard RGB images, concluding that modern RGB cameras are already sufficient if processed appropriately (Gutiérrez et al., 2019). Satellite approaches have been less successful: mango yield prediction from very-high-resolution imagery obtained 26.5% error at the individual tree level, concluding that no universal relationship exists between satellite vegetation indices and fruit counts (Torgbor et al., 2024).

Double counting in video has been addressed by combining YOLOv4-tiny with Kalman filters to count apples in video sequences, reaching 94.47% detection precision and 81.94% counting accuracy, with a coefficient of determination of 0.986 relative to manual counting (Gao et al., 2021). This line was later extended with Cascade-SORT, fusing motion and appearance features through Kalman filters and Mahalanobis distances, obtaining lower errors than standard SORT in camellia and apple videos (He et al., 2022). A systematic comparison of tracking-by-detection algorithms for apple orchard production estimation concluded that the combination of a YOLO detector with DeepSORT was the most robust (Villacrés et al., 2023).

Fruit detection methods have been applied to a range of crops. Green oranges were counted directly from trees using artificial intelligence, with detection counts closely matching manual references (Gremes et al., 2023). Faster R-CNN was adapted for strawberries, replacing the backbone with a multi-cascade network and RoiPooling with RoiAlign, obtaining average precisions of 0.9019 for ripe and 0.8447 for unripe fruit (Li et al., 2023). A particularly relevant challenge for tomato cultivation was addressed by adding illumination and planting-density variability through domain adaptive learning, reaching 96.80% mAP and exceeding YOLOv8 by 2.17 points (Li et al., 2024). Maturity recognition and fruit counting for sweet peppers in greenhouses achieved 85.7% precision under difficult conditions (Viveros Escamilla et al., 2024), which constitutes the closest antecedent to the category classification task addressed here. In addition, a lightweight pear detector based on frequency domain data augmentation and YOLOv7 reached 97.23% mAP at 69.39 frames per second (Zheng and Yang, 2024).

Peru-specific contributions have also emerged in recent years. In Lambayeque, a system for the automated detection and counting of Gossypium barbadense fruits achieved precisions close to 100% and was integrated into a web application accessible to growers (Ballena-Ruiz et al., 2025). In Tacna, several deep learning models were evaluated for olive detection and counting, concluding that the YOLO family was the most effective, with 94.96% precision and a coefficient of determination of 0.944 (Osco-Mamani et al., 2025). These works demonstrate that detection technologies can be adapted to Peruvian conditions, but also that models do not transfer directly: a detector trained in another country or crop requires retraining with local data.

This review allows several research gaps to be identified. First, YOLO architectures dominate the detection field owing to their balance between speed and accuracy, but region-based detectors remain a reference when accuracy takes precedence over speed. Second, almost all studies confine themselves to detecting and counting; very few evaluate classification into commercial fruit categories, which is precisely the information that determines the economic value of the harvest. Third, most mango studies were conducted in Australia, China and India, and the few Peruvian contributions address other crops, none addressing counting by commercial categorization. Fourth, models do not transfer directly, since climate, tree architecture and fruit morphology differ between production regions, so that collecting local data, retraining and validating is indispensable. Finally, correction for hidden fruit becomes unavoidable if actual production, and not merely visible fruit, is to be estimated with greater accuracy.

Accordingly, the present research addresses the following question: is it possible to optimize the estimation of Kent mango production by commercial category using machine learning techniques combined with image analysis? The general objective was to develop a method based on machine learning and image processing to estimate the production of Kent mango fruit by export category, implementing deep learning models that detect and classify fruit in RGB images obtained under real field conditions in the Motupe district. The specific objectives were: (i) to build a set of RGB images of Kent mango in Motupe fields, defining the commercial export categories as classification classes; (ii) to preprocess and label the dataset, establishing an annotation protocol that distinguishes categories by colour, size and ripeness according to market standards; (iii) to implement and train detection and classification architectures, evaluating precision, recall, mean average precision and processing time; and (iv) to validate the method by comparing automated estimates against manual estimates.

## 2 Materials and methods

### 2.1 Study design

The research is applied in nature, since it is oriented towards solving a concrete problem in the agricultural sector, namely the estimation of Kent mango production by export category. The design is quasi-experimental: five object detection models were trained and compared under controlled conditions, holding constant the main hyperparameters, the dataset partition and the random seed across all experiments. The design qualifies as quasi-experimental rather than fully experimental because the detector architecture, the manipulated factor, could not be randomly assigned to the fixed pool of annotated images; instead, every architecture was evaluated on the same pre-existing training, validation and test partitions, which functioned as matched, non-randomized comparison groups. The only factor varying between experiments is the detector architecture, which allows the observed differences in the metrics to be attributed to the model and not to the training conditions.

### 2.2 Study area and image acquisition

The study population comprised Kent mango (*Mangifera indica* L., cv. Kent) farms located in the Motupe district, province and department of Lambayeque, northern Peru. Ten farms at the harvest stage were selected and 20 sample trees were chosen from each, including trees of different sizes and fruit loads, giving a total of 200 evaluated trees. Sampling was non-probabilistic and by convenience, conditioned by access to the farms and by coincidence with the harvest calendar. **Figure 1** shows the location of the region within the national territory and the distribution of the farms from their GPS coordinates.

**Figure 1:**
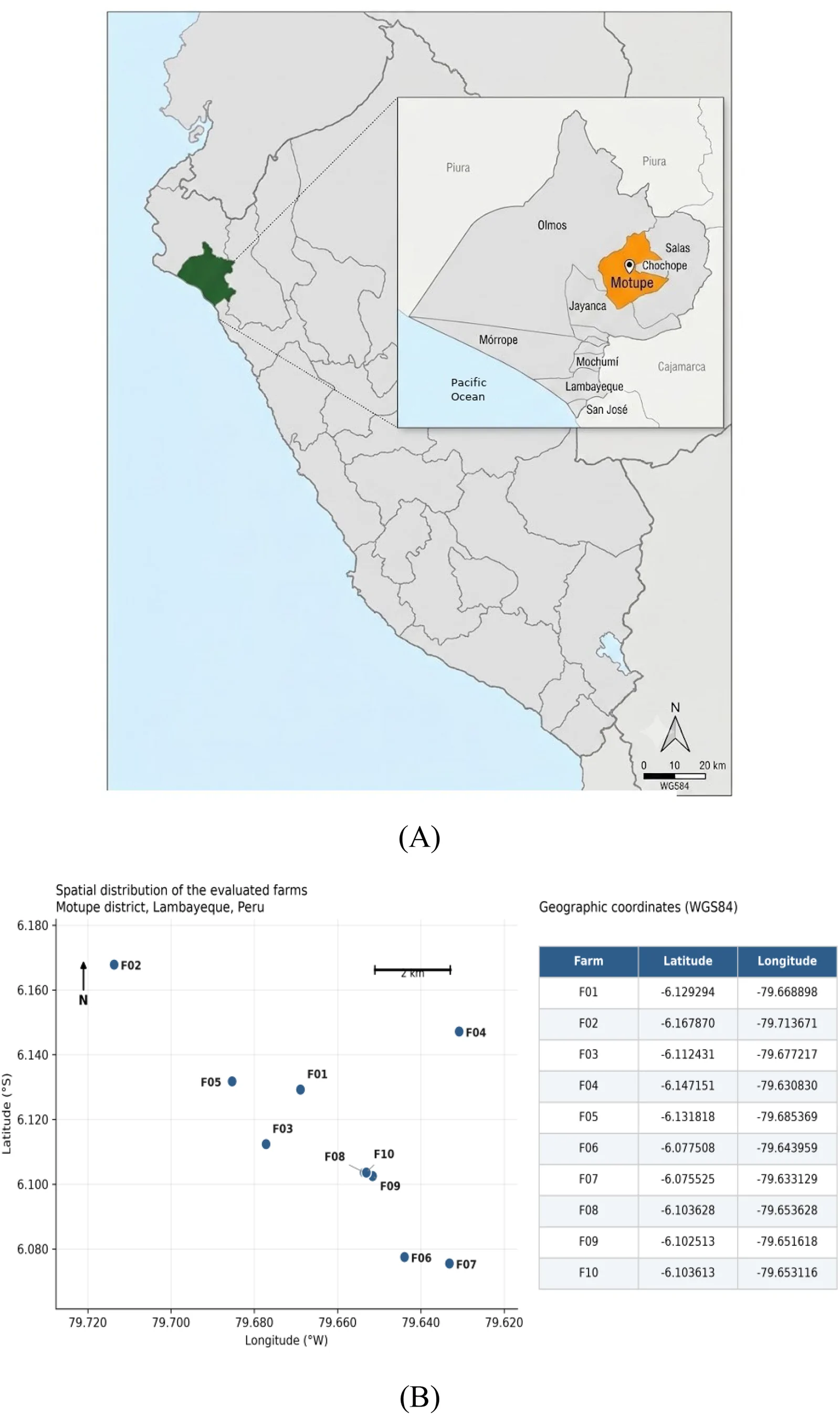
Geographic location of the evaluated farms: **(A)** Lambayeque region within Peru; **(B)** distribution of the ten farms in the Motupe district.

**Table 1** details the characterization of the ten farms, with their geographic coordinates and the harvested production by category. Total harvested production across the ten farms amounted to 345,649 kilograms, of which 98.7% corresponded to the sea freight category and 1.3% to the air freight category. Reject fruit is not accounted for as harvested production, since it is not destined for export trade. The air freight category was present in only four of the ten evaluated farms, which confirms the scarcity of fruit of optimal calibre for air freight export.

**Table 1:** Characterisation of the evaluated farms and harvested production by category.

Farm	Date	Latitude	Longitude	Plants	Air freight (kg)	Sea freight (kg)	Total harvested (kg)
01	18/01/2025	-6.129294	-79.668898	7,500	0	86,189	86,189
02	18/01/2025	-6.167870	-79.713671	2,500	2,394	112,230	114,624
03	20/01/2025	-6.112431	-79.677217	500	685	27,655	28,340
04	21/01/2025	-6.147151	-79.630830	1,200	0	27,722	27,722
05	21/01/2025	-6.131818	-79.685369	500	1,048	19,173	20,221
06	22/01/2025	-6.077508	-79.643959	600	403	10,148	10,551
07	22/01/2025	-6.075525	-79.633129	550	0	33,323	33,323
08	24/01/2025	-6.103628	-79.653628	130	0	3,401	3,401
09	24/01/2025	-6.102513	-79.651618	450	0	11,774	11,774
10	24/01/2025	-6.103613	-79.653116	400	0	9,504	9,504
Total				14,330	4,530	341,119	345,649

Images were captured with a Samsung Galaxy A52 mobile device in JPG format with RGB encoding and a resolution of 3,468 × 4,624 pixels. Photographs were taken between 08:00 and 13:00, the period during which field evaluation staff begin their visual and manual production estimation work. The photographer stood in front of each selected tree at a distance of approximately 7 to 8 metres, reducing the distance when the plant was smaller. The device was held at the operator’s face height, approximately 1.70 metres, always maintaining the vertical orientation of the phone. Two images were captured per tree, one from the front and one from the rear relative to the first shot, mirroring the evaluation performed by specialist personnel. This two-view capture replicates the multi-view logic that has been shown to reduce underestimation from occlusion in mango orchards (Anderson et al., 2021; Sarron et al., 2023). An initial set of 443 photographs was obtained, which was subsequently filtered to the 400 final images used for training. **Figure 2** shows two representative examples of the captured image set.

**Figure 2:**
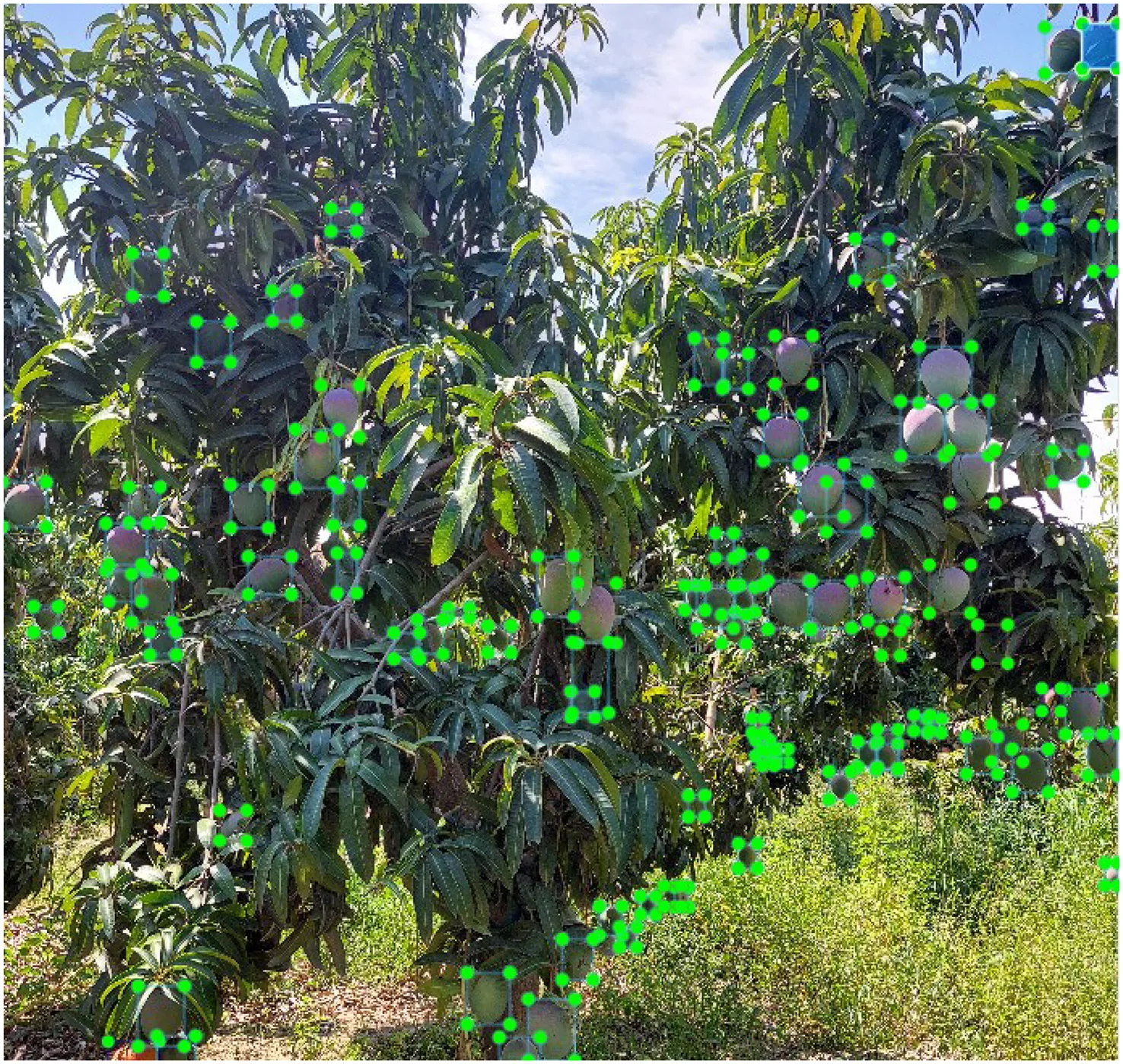
Representative sample of the Kent mango dataset captured in the field (front and rear view of the same tree).

### 2.3 Annotation protocol and class definition

Dataset labelling was performed with LabelImg version 1.8.6, open source software under the MIT licence that allows bounding boxes to be drawn visually and annotations to be exported in YOLO format. Each fruit was labelled with one of three categories, air freight, sea freight or reject, following the classification criteria used by the processing plant for Kent mango export. **Table 2** describes the assignment criteria for each category. Visible fruit were annotated with bounding boxes fitted to the observable contour. Partially occluded fruit were annotated when the visible portion allowed the annotator to delimit a box and assign a commercial category consistently; completely hidden or visually indistinguishable fruit were not annotated. A dataset-level quality control pass checked class indices, YOLO coordinate format, empty labels, and consistency of the train/validation/test class distributions.

**Table 2:** Classification criteria by commercial export category.

Class index	Category	Commercial destination	Assignment criterion
0	Air freight	Air freight export	Fruit of optimal calibre and colouring, without visible defects in the epidermis; corresponds to the category of highest commercial value.
1	Sea freight	Sea freight export	Fruit suitable for export with longer transit time; admits a lower degree of ripeness and slight imperfections that do not compromise conservation.
2	Reject	Domestic market/industry	Fruit with mechanical damage, pest damage, deformation or calibre outside specification; does not meet export requirements.

The YOLO annotation format represents each object through a text line containing the class and the normalized coordinates of the box centre, together with its width and height, as shown in Equation 1:

(1) $$\left(\text{class},\;x_{c}/W,\;y_{c}/H,\;w/W,\;h/H\right)$$

where x_c and y_c are the coordinates of the bounding box centre in pixels, w and h its width and height, and W and H the dimensions of the image. Normalization makes the annotation independent of resolution, which is necessary because the images are rescaled during training.

The dataset comprises a total of 66,465 annotated instances distributed markedly unevenly across the three categories: 57,213 instances of the sea freight class (86.1%), 6,780 of the reject class (10.2%) and only 2,472 of the air freight class (3.7%). The imbalance is severe: the majority class exceeds the air freight class by a factor of 23.1 and the reject class by a factor of 8.4. This condition is inherent to the phenomenon under study and not to a sampling bias, since it reflects the actual composition of production, in which fruit of optimal quality for air freight export constitutes a minority fraction of the total harvest. The proportion of air freight category fruit varies considerably between farms, ranging from 14.0% in farm F01 to 0.2% in F07.

### 2.4 Patch generation and dataset partitioning

Since the original images have a high resolution (3,468 × 4,624 pixels) and the fruits appear as small objects, direct rescaling to the network input resolution would cause many fruits to occupy only a few pixels, hindering their detection. Each image was therefore divided into patches of 1,280 × 1,280 pixels with an overlap of 20%. This strategy increases the number of training instances, preserves the effective resolution of each fruit and helps the model learn local patterns. The displacement step between consecutive patches is obtained as shown in Equation 2:

(2) s=p·(1−o)

where p is the patch size and o the overlap fraction, giving a step of 1,024 pixels. The overlap guarantees that fruit located at the border of a patch appear complete in the adjacent patch, avoiding truncated boxes. The number of patches per image is determined as shown in Equation 3:

(3) N=([(W−p)/s]+1)·([(H−p)/s]+1)

**Figure 3** summarizes the pipeline of the proposed method.

**Figure 3:**
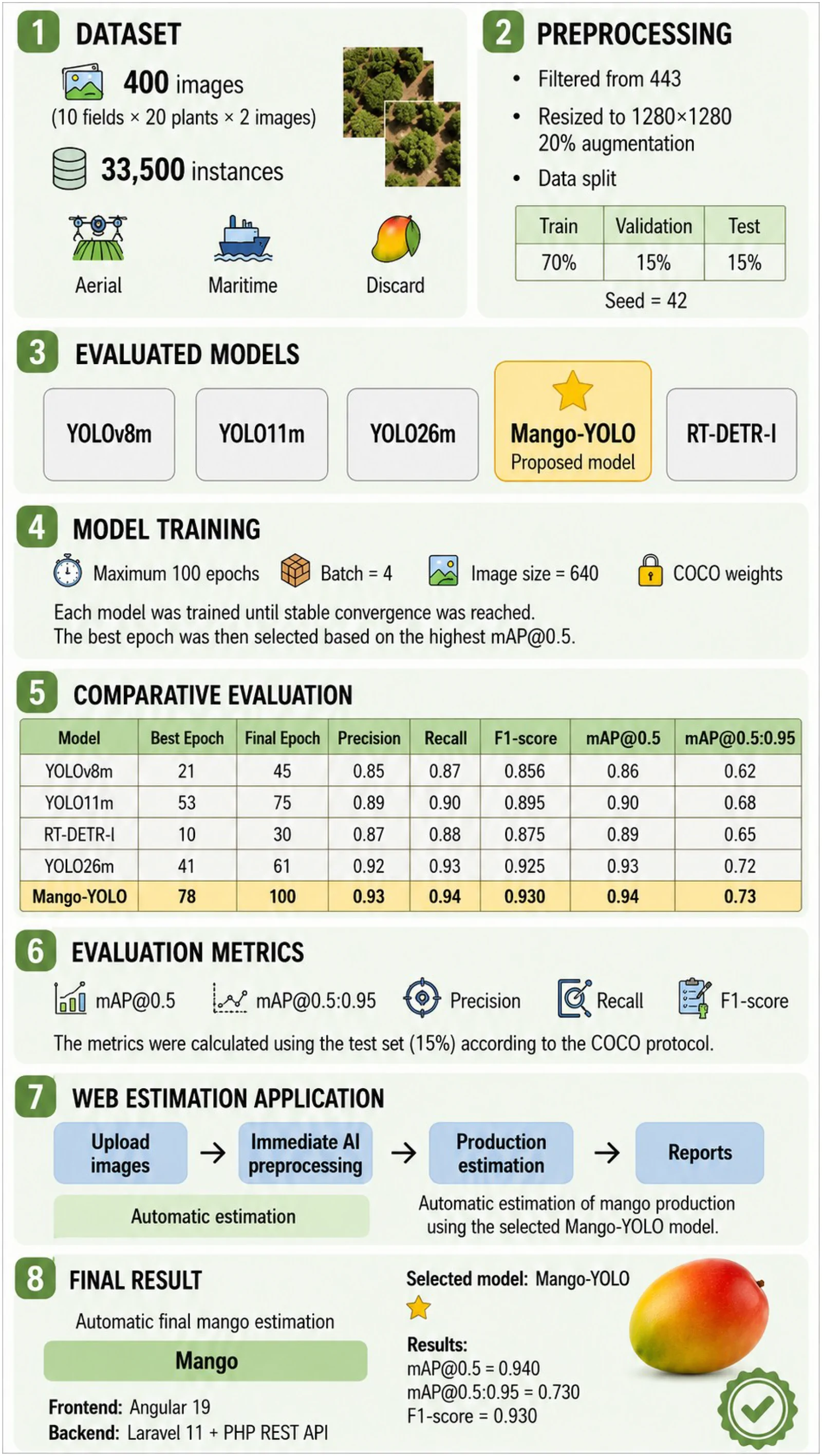
General pipeline of the proposed method for estimating Kent mango production by category.

Each patch retained the YOLO annotations of the fruits contained within its area, recalculating the box coordinates relative to the new origin. Two filtering criteria were applied to guarantee the quality of the resulting annotations. The first establishes a minimum of one instance per patch, so that patches corresponding to foliage or sky regions without fruit are discarded and do not introduce empty examples into training. The second sets a minimum area ratio of 0.30, meaning that a bounding box sectioned by the patch border is retained only if at least 30% of its original area remains inside it; otherwise it is removed, preventing the model from learning from non-representative fruit fragments. **Figure 4** illustrates the patch generation process.

**Figure 4:**
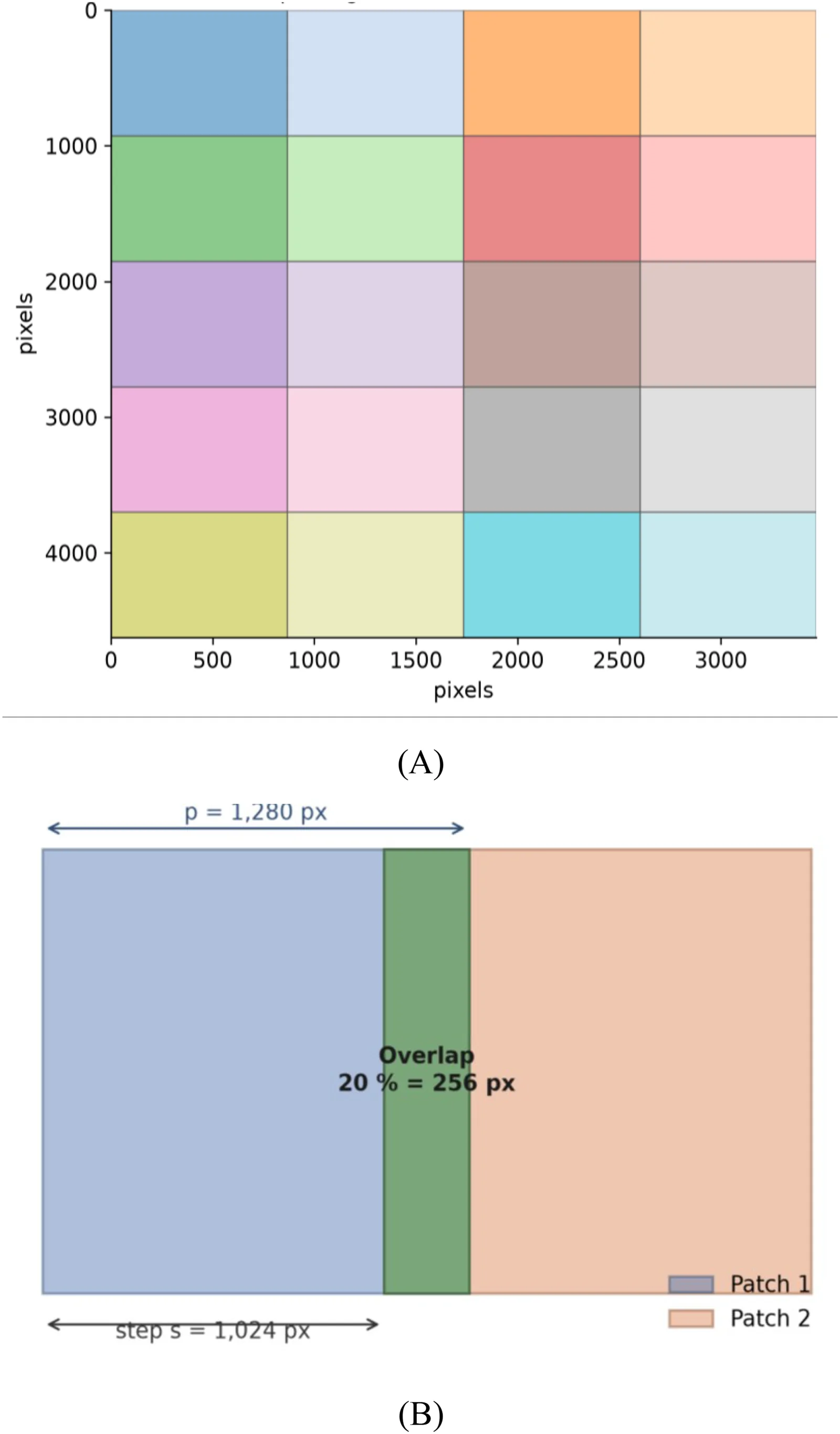
Patch generation process: **(A)** division of the original image into a patch grid; **(B)** 20% overlap between adjacent patches.

The resulting set comprised 6,544 patches containing a total of 66,465 annotated fruit instances. The partition was performed in a 70%/15%/15% proportion for training, validation and testing, using a random seed of 42 to guarantee reproducibility. The seed was fixed simultaneously in the random number generators of Python, NumPy and PyTorch, and the deterministic mode of the latter was enabled. **Table 3** details the distribution by partition, where instance density remains homogeneous across the three partitions, with a global mean of 10.16 fruits per patch. This homogeneity confirms that the stratified random partition did not introduce a density bias between the training, validation and test sets, and that class proportions remain stable with deviations below one percentage point. Given that patch generation already multiplies the number of instances per class considerably, no additional oversampling technique was applied to the minority classes.

**Table 3:** Distribution of the patch dataset by partition.

Partition	Patches	% of patches	Instances	% of instances	Instances per patch	% Air freight	% Sea freight	% Reject
Training	4,557	69.6%	45,915	69.1%	10.08	3.9%	85.8%	10.3%
Validation	968	14.8%	9,595	14.4%	9.91	2.6%	87.8%	9.7%
Test	1,019	15.6%	10,955	16.5%	10.75	4.0%	85.6%	10.4%
Total	6,544	100.0%	66,465	100.0%	10.16	**3.7%**	**86.1%**	**10.2%**

Bold values indicate the overall totals and percentages for the complete dataset.

### 2.5 Detection models evaluated

Four baseline models were selected at the m scale, corresponding to the medium size of each family, plus the proposed architecture. The choice of the m scale sought a balance between representational capacity and computational cost, so that the models could be trained on the available GPU and subsequently deployed on the web application server. **Table 4** compares the characteristics of each model.

**Table 4:** Detection models evaluated and their main characteristics.

Model	Family/paradigm	Distinctive characteristic	Reference
YOLOv8m	One-stage anchor-free CNN	Decoupled detection head; balance between speed and accuracy.	Arcila-Díaz et al., 2024
YOLO11m	One-stage anchor-free CNN	C3k2, SPPF and C2PSA blocks for more efficient feature extraction.	Liao et al., 2025
YOLO26m	One-stage anchor-free CNN	NMS-free inference; removes Distribution Focal Loss; label assignment oriented to small objects.	Jocher et al., 2026
RT-DETR-l	Transformer (real-time DETR)	Efficient hybrid encoder; no NMS required; adjustable inference speed.	Zhao et al., 2024
Mango-YOLO (proposed)	One-stage CNN + attention	YOLO26m as base, with CBAM, BiFPN and MPDIoU loss.	Woo et al., 2018; Tan et al., 2020; Ma and Xu, 2023

### 2.6 Proposed architecture: Mango-YOLO

The Mango-YOLO architecture is built on the YOLO26m structure and incorporates three modifications targeting the specific characteristics of the problem: small fruit, partial occlusion by foliage and high visual similarity between the air freight and sea freight categories. The modifications are a Convolutional Block Attention Module (CBAM) at the end of the backbone, a neck based on BiFPN nodes, and the replacement of the regression loss function by MPDIoU.

The complete layer-by-layer configuration of Mango-YOLO, including the input source, module type, main arguments and functional purpose of each layer, is provided in **Table 5**.

**Table 5:** Layer-by-layer configuration of the proposed Mango-YOLO architecture.

Layer	From	Module	Arguments	Purpose
0	-1	Conv	[64, 3, 2]	Initial downsampling and low-level feature extraction.
1	-1	Conv	[128, 3, 2]	Second downsampling stage for early spatial features.
2	-1	C3k2 x2	[256, False, 0.25]	CSP feature extraction with compact bottleneck blocks.
3	-1	Conv	[256, 3, 2]	Downsampling toward the P3 feature scale.
4	-1	C3k2 x2	[512, False, 0.25]	Intermediate feature extraction for small and medium fruit.
5	-1	Conv	[512, 3, 2]	Downsampling toward the P4 feature scale.
6	-1	C3k2 x2	[512, True]	Deeper backbone representation with shortcut connections.
7	-1	Conv	[1024, 3, 2]	Downsampling toward the P5 semantic feature scale.
8	-1	C3k2 x2	[1024, True]	High-level semantic feature extraction.
9	-1	SPPF	[1024, 5, 3, True]	Spatial pyramid pooling to enlarge receptive field.
10	-1	C2PSA	[1024]	Position-sensitive attention before the proposed attention block.
11	-1	CBAM	[512]	Channel and spatial attention to emphasise visible mango regions.
12	[11, 6]	BiFPN_Node	[512, 256, 256]	Top-down fusion from P5 and P4 features.
13	[-1, 4]	BiFPN_Node	[256, 128, 128]	Top-down fusion to obtain the P3 detection feature.
14	[-1, 12]	BiFPN_Node	[128, 256, 256]	Bottom-up fusion to refine the P4 detection feature.
15	[-1, 11]	BiFPN_Node	[256, 512, 512]	Bottom-up fusion to refine the P5 detection feature.
16	[13, 14, 15]	Detect	[3]	Three-class detection head for air freight, sea freight and reject fruit.

The Convolutional Block Attention Module refines the feature map by sequentially applying channel attention and spatial attention (Woo et al., 2018). Channel attention determines which channels are most informative, and spatial attention determines in which regions of the image to concentrate the response. Given an input feature map F, channel attention is computed as shown in Equation 4:

(4) $$M_{c}\left(F\right)=\sigma \left(MLP\left(AvgPool\left(F\right)\right)+MLP\left(MaxPool\left(F\right)\right)\right)$$

where σ is the sigmoid function and MLP is a shared multilayer perceptron with a hidden reduction layer of r = 16. Spatial attention is then obtained as shown in Equation 5:

(5) $$M_{s}\left(F\right)=\sigma \left(f^{7\times 7}\left(\left[AvgPool\left(F\right);MaxPool\left(F\right)\right]\right)\right)$$

where f^{7×7} denotes a convolution with a 7 × 7 kernel and the pooling operators are applied along the channel dimension. The refined output map is obtained by element-wise product, as shown in Equation 6:

(6) $$F\prime =M_{c}\left(F\right)⊗F,\;F^{\prime \prime }=M_{s}\left(F\prime \right)⊗F\prime$$

**Figure 5** summarizes the data flow through the CBAM module.

**Figure 5:**
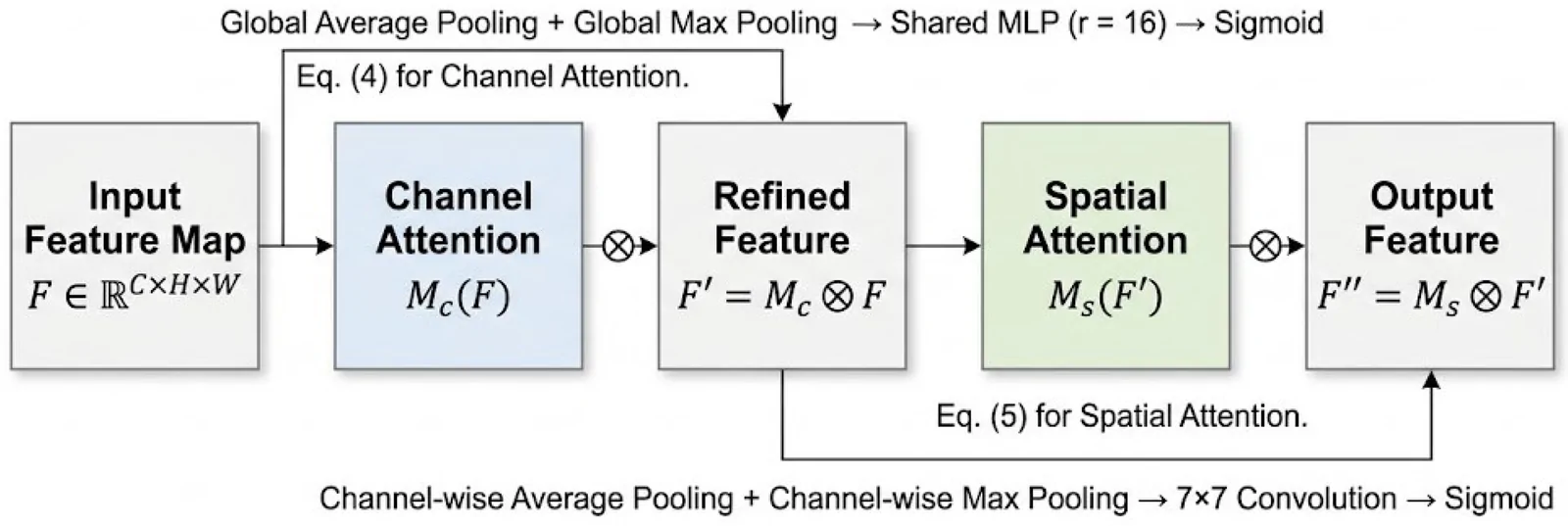
Data flow of the CBAM attention module, with its channel attention and spatial attention submodules.

The weighted bidirectional feature pyramid network, proposed in EfficientDet, replaces the simple concatenation fusion used in conventional necks (Tan et al., 2020). Each BiFPN node projects the inputs to a common number of channels, interpolates the maps to a common resolution and combines them through a weighted sum with learned weights. Fast normalized fusion is defined as shown in Equation 7:

(7) $$O=\left(\sum w_{i}\cdot I_{i}\right)/\left(\epsilon +\sum w_{j}\right)$$

where I_i are the input maps, w_i the learned weights constrained to non-negative values through ReLU, and ϵ = 10^−6^ a numerical stability term that avoids division by zero. This weighting allows the network to assign greater weight to the scales that are most informative for the dataset, instead of treating all scales equally. In the implemented model, four BiFPN nodes build the top-down and bottom-up paths of the pyramid, producing the three output maps P3, P4 and P5 that feed the detection head.

The MPDIoU loss explicitly incorporates the distance between the extreme points of the predicted box and the reference box, improving regression convergence when objects are small or overlap (Ma and Xu, 2023). The standard intersection over union is first computed as shown in Equation 8:

(8) $$IoU=\left|B\cap B_{gt}\right|/\left|B\cup B_{gt}\right|$$

where B and B_gt are the predicted and reference boxes. The squared Euclidean distances between the top-left and bottom-right corners of both boxes are then computed as shown in Equations 9 and 10:

(9) $$d_{1}^{2}={\left(x_{1}-x_{1,gt}\right)}^{2}+{\left(y_{1}-y_{1,gt}\right)}^{2}$$

(10) $$d_{2}^{2}={\left(x_{2}-x_{2,gt}\right)}^{2}+{\left(y_{2}-y_{2,gt}\right)}^{2}$$

Both distances are normalized with respect to the diagonal of the reference box, and the loss is defined as shown in Equation 11:

(11) $$L_{MPDIoU}=1-IoU+d_{1}^{2}/\left(w_{gt}^{2}+h_{gt}^{2}\right)+d_{2}^{2}/\left(w_{gt}^{2}+h_{gt}^{2}\right)$$

where w_gt and h_gt are the width and height of the reference box. Normalization by the diagonal makes the penalty invariant to object scale, which is relevant in this dataset, where the apparent size of the fruit varies with the distance from the tree to the camera. In the implementation, the loss was integrated by substituting the IoU computation within the Ultralytics metrics module.

### 2.7 Training configuration

All models were trained for a maximum of 100 epochs with a batch size of 4 and an input resolution of 640 × 640 pixels; the 1,280 × 1,280 patches are rescaled to that resolution within the data loader. The random seed was fixed at 42 and deterministic mode was enabled to guarantee reproducibility. Early stopping was used with a patience of 20 epochs, so that training halted automatically if no improvement was observed in the validation metric during that period; the retained checkpoint always corresponds to the best epoch and not to the last. The YOLO and RT-DETR-l models were initialized with weights pretrained on the COCO dataset, applying transfer learning (Pan and Yang, 2010). Data augmentation techniques were applied during training (Shorten and Khoshgoftaar, 2019), whose values are detailed in **Table 6**. The data augmentation configuration of Mango-YOLO is deliberately more aggressive than that of the baselines, because the proposed architecture is trained from a configuration defined by a YAML file and not from pretrained weights, and therefore requires greater apparent variability in the data to compensate for the absence of COCO initialization and to reduce the risk of overfitting.

**Table 6:** Training and data augmentation hyperparameters.

Hyperparameter	Baselines (YOLOv8m, YOLO11m, YOLO26m)	Mango-YOLO	RT-DETR-l
Epochs (maximum)	100	100	100
Batch size	4	4	4
Input resolution	640 × 640	640 × 640	640 × 640
Random seed	42	42	42
Patience (early stopping)	20	20	20
Optimiser	Automatic (AdamW)	AdamW	Automatic
Initial learning rate (lr0)	0.01	0.01	0.01
Final learning rate (lrf)	0.01	0.01	0.01
Dropout	0.1	0.1	n/a
Mosaic	1.0	1.0	n/a
Mixup	0.1	0.2	n/a
Hue variation (hsv_h)	0.015	0.02	n/a
Saturation variation (hsv_s)	0.7	0.8	n/a
Brightness variation (hsv_v)	0.4	0.5	n/a
Rotation (degrees)	5	10	n/a
Translation	0.1	0.15	n/a
Scaling	0.5	0.6	n/a
Horizontal flip (fliplr)	0.5	0.5	n/a
Vertical flip (flipud)	n/a	0.1	n/a

Training was executed on Google Colaboratory through a Colab Pro subscription, which provided access to an NVIDIA L4 graphics processing unit with 22 GB of VRAM, 4 virtual CPUs and 53 GB of system RAM, with mixed precision (AMP) enabled by Ultralytics. The software environment comprised Python 3.12, PyTorch 2.11 with CUDA 12.8, Ultralytics 8.4.87, NumPy and scikit-learn.

### 2.8 Evaluation metrics

Model evaluation was carried out on the validation set during training and subsequently on the test set, which took no part in any phase of the fitting. A detection is considered a true positive (TP) when the intersection over union with a reference box of the same class exceeds an established threshold; otherwise it is considered a false positive (FP). Reference boxes with no associated detection constitute false negatives (FN). Precision is defined as shown in Equation 12:

(12) P=TP/(TP+FP)

recall as shown in Equation 13:

(13) R=TP/(TP+FN)

and the F1-score, the harmonic mean of both, as shown in Equation 14:

(14) F1=2·(P·R)/(P+R)

The average precision (AP) of a class is obtained as the area under the precision-recall curve, computed through 101-point interpolation, as shown in Equation 15:

(15) $$AP=\left(1/101\right)\cdot \sum P_{interp}\left(r\right),\;r\in \left\{0,0.01,\ldots ,1\right\}$$

The mean average precision (mAP) is the average of the AP over the three classes of the problem, as shown in Equation 16:

(16) $$mAP=\left(1/K\right)\cdot \sum AP_{k},\;k=1,\ldots ,K,K=3$$

Two variants are reported. The mAP@0.5 metric uses a single IoU threshold of 0.50 and fundamentally evaluates classification and detection capability. The mAP@0.5:0.95 metric averages the mAP computed over ten equally spaced IoU thresholds between 0.50 and 0.95, as shown in Equation 17, and is considerably more demanding, since it penalizes imprecise bounding box adjustments:

(17) $$mAP@0.5:0.95=\left(1/10\right)\cdot \sum mAP_{@t},\;t\in \left\{0.50,\ldots ,0.95\right\}$$

### 2.9 Yield estimation and web application

Once the best detection model had been selected, production per category was estimated. For each photograph, detections classified as air freight, sea freight or reject are counted. Since only a sample of the farm’s plants is photographed, the resulting count must be scaled to the total number of plants through the factor shown in Equation 18:

(18) $$k=N_{total}/N_{sampled}$$

and the scaled count of each category c is obtained as shown in Equation 19:

(19) $${\hat{n}}_{c}=n_{c}\cdot k$$

Yield in kilograms was estimated from the automatic count by commercial category using Monte Carlo simulation. For each category c, the system used a mean fruit weight μ_c and a standard deviation σ_c as conversion parameters. The mean weights used in the projection were 0.45 kg for air freight, 0.40 kg for sea freight and 0.35 kg for reject fruit; these values were kept fixed across all evaluated farms to ensure comparability between validation units. S = 10,000 simulations are generated; in each simulation s, the weight of each of the ${\hat{n}}_{c}$ fruits is sampled independently from a normal distribution, as shown in Equation 20:

(20) $$w_{i}\sim N\left(\mu_{c},\sigma_{c}^{2}\right),\;i\;=\;1,\ldots ,{\hat{n}}_{c}$$

The total weight of the category in simulation s is the sum of the individual weights, and the total weight of the farm is the sum over the three categories, as shown in Equation 21:

(21) $$W_{s}=\sum_{c}\sum_{i=1}{\hat{n}}_{c}w_{i,c}$$

The point estimate of production is the mean of the set of simulations, as shown in Equation 22:

(22) $$\hat{Y}=\left(1/S\right)\cdot \sum W_{s},\;s=1,\ldots ,S$$

and the 90% confidence interval is obtained directly from the empirical percentiles of the simulated distribution, as shown in Equation 23:

(23) $$CI_{90\%}=\left[P_{5}\left(W\right),\;P_{95}\left(W\right)\right]$$

When the standard deviation of a category is not recorded or is zero, the system assumes by default σ_c = 0.10 · μ_c.

To propagate uncertainty in the category mean weight, the system included a parametric simulation variant in which μ_c is treated as an operational random variable. In each iteration, a possible value of μ_c is first sampled from a normal distribution, as shown in Equation 24:

(24) $$\mu_{c}\sim N\left(\mu_{0},\tau^{2}\right)$$

In each of the M = 10,000 samples, a value of the mean weight is first drawn from the posterior distribution and, conditional on that value, the individual weights of the fruits are generated, as shown in Equation 25:

(25) $$w_{i}|\mu_{c}\sim N\left(\mu_{c},\sigma_{c}^{2}\right)$$

Total production is obtained by aggregation as in the Monte Carlo procedure, and the 90% interval is computed from the empirical percentiles of the simulated distribution. Accordingly, the implementation is described as Monte Carlo simulation with parametric uncertainty in the mean fruit weight by category.

The web application, named Mango Smart, allows users to upload photographs of Kent mango plantations and obtain an estimate of production by category. It is structured in a three-tier client-server architecture. The presentation layer, developed in Angular 19, runs in the user’s browser and manages photograph upload and result visualization, compressing each image to a maximum of 1,920 × 1,080 pixels at 85% JPEG quality before sending it. The business logic layer, implemented in Laravel on PHP 8.2, exposes a REST API that orchestrates the flow, stores the images, invokes the Python inference and estimation scripts, and persists the results. The data layer, on SQL Server, stores the estimates, the photographs and the categories. The inference engine uses Python and Ultralytics to run the Mango-YOLO model, whereas the estimation engine uses Python and NumPy to convert fruit counts into kilograms through Monte Carlo simulation. **Figure 6** presents this architecture.

**Figure 6:**
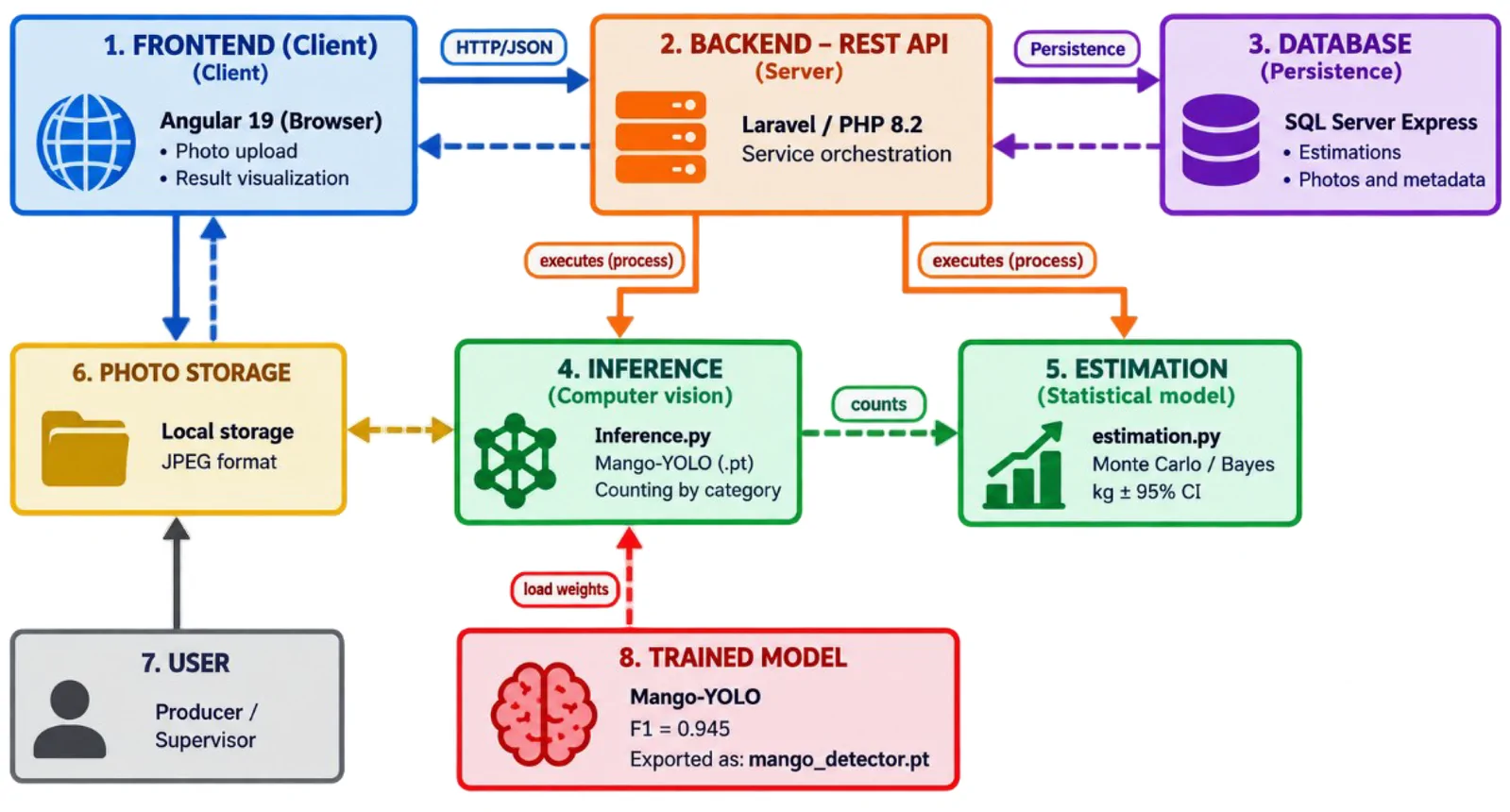
Architecture of the Mango Smart system, with its three layers and the inference and estimation engines.

## 3 Results

### 3.1 Overall detection performance

The models were trained under identical conditions and evaluated on the test set, composed of 1,019 patches with 10,955 annotated instances that took no part in weight fitting. **Table 7** presents the consolidated results matrix, which constitutes the basis of all analyses in this section. Two derived columns are incorporated: the localisation gap, defined as the difference between mAP@0.5 and mAP@0.5:0.95, and the number of epochs elapsed after reaching the optimum, which evidences the activation of the early stopping mechanism.

**Table 7:** Comparative performance of the detection models on the test set.

Model	Best epoch	Last epoch	Epochs after optimum	Precision	Recall	F1-score	mAP@0.5	mAP@0.5:0.95	mAP gap
YOLOv8m	21	45	24	0.860	0.870	0.865	0.860	0.620	0.240
YOLO11m	55	75	20	0.890	0.900	0.895	0.900	0.680	0.220
YOLO26m	41	61	20	0.920	0.930	0.925	0.930	0.720	0.210
RT-DETR-l	10	30	20	0.870	0.880	0.875	0.880	0.650	0.230
Mango-YOLO	78	100	22	0.930	0.940	0.930	0.940	0.730	0.210

The Mango-YOLO model obtained the best performance across the five evaluated metrics, reaching its optimum at epoch 78 within the 100-epoch budget assigned to all experiments, with a precision of 0.930, a recall of 0.940, an F1-score of 0.930, a mAP@0.5 of 0.940 and a mAP@0.5:0.95 of 0.730. It was followed by YOLO26m with an F1-score of 0.925 and a mAP@0.5 of 0.930, and then YOLO11m, with an F1-score of 0.895 and a mAP@0.5 of 0.900. RT-DETR-l occupied fourth position with an F1-score of 0.875, ahead of YOLOv8m, which recorded 0.865 and obtained the lowest performance of the set, with a mAP@0.5:0.95 of 0.620. **Figure 7** allows the four main metrics to be compared visually across the five evaluated models.

**Figure 7:**
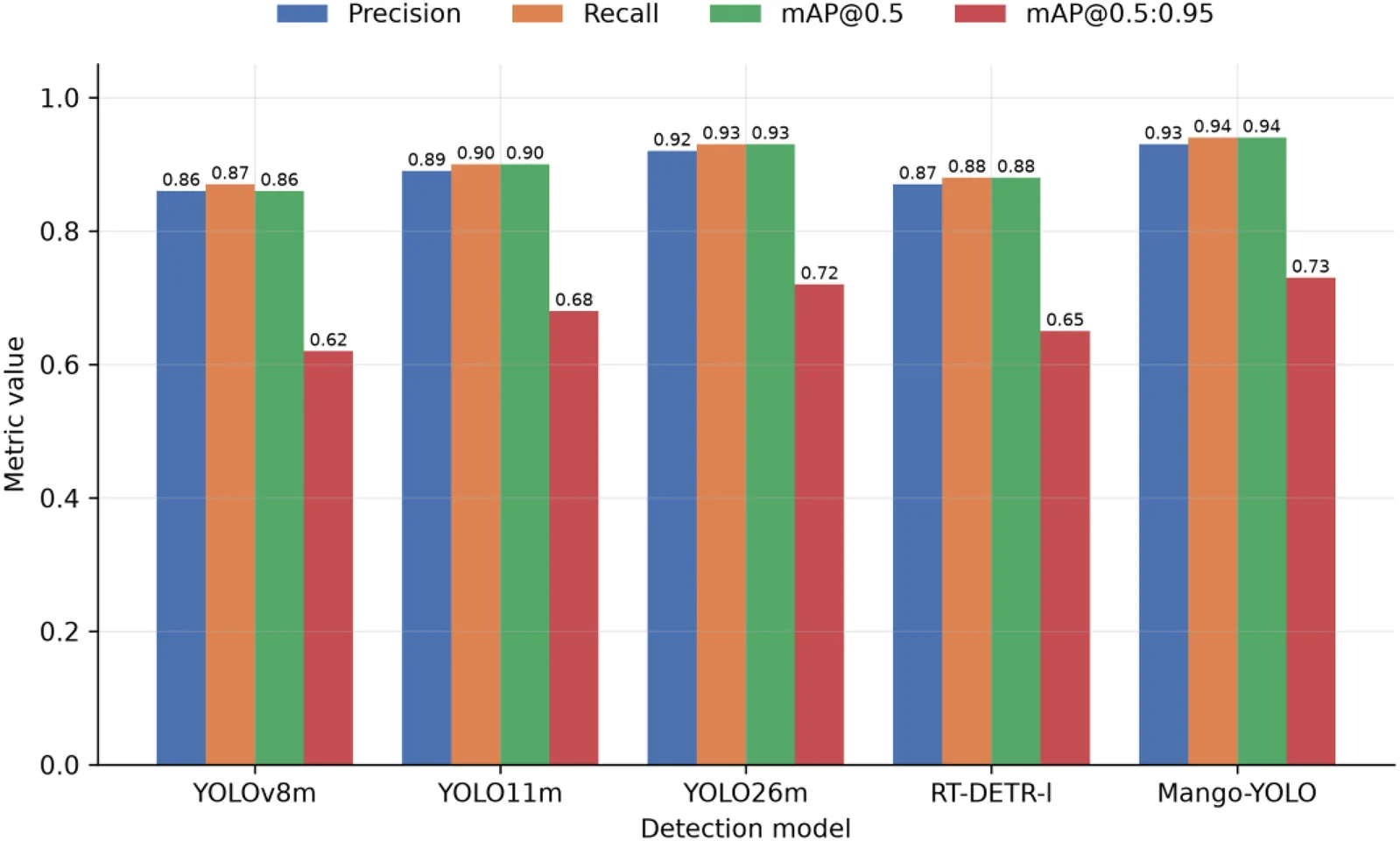
Comparison of precision, recall, mAP@0.5 and mAP@0.5:0.95 across the five evaluated models.

To quantify the magnitude of each architecture’s advantage over the others, a pairwise difference matrix of the F1-score was constructed, whose element in row i and column j corresponds to the difference between the F1-score of model i and that of model j, as shown in Equation 26:

(26) $$D_{ij}=F1_{i}-F1_{j}$$

Positive values indicate that the row model outperforms the column model, and negative values the opposite. The row corresponding to Mango-YOLO presents positive values in all its cells, confirming that this model outperforms the four remaining architectures without exception, while the row for YOLOv8m presents negative values throughout, being the lowest-performing model of the set. The difference between the best and the worst model is 0.065 F1-score points (0.930 against 0.865), representing a 7.5% gap in relative terms. The distance between Mango-YOLO and YOLO26m, the two best models, is 0.005 points, while both are separated from YOLO11m, the third-ranked model, by 0.035 and 0.030 points respectively. This structure reveals that the proposed architecture and its base model form a performance block clearly separated from the three remaining architectures, among which differences do not exceed 0.030 points.

### 3.2 Training convergence

The training log of each model allows the evolution of the metrics across epochs to be analyzed beyond the final consolidated value. **Figure 8** presents the evolution of mAP@0.5:0.95 on the validation set; the circular marker indicates the epoch at which each model reached its optimum, that is, the checkpoint retained for the final evaluation.

**Figure 8:**
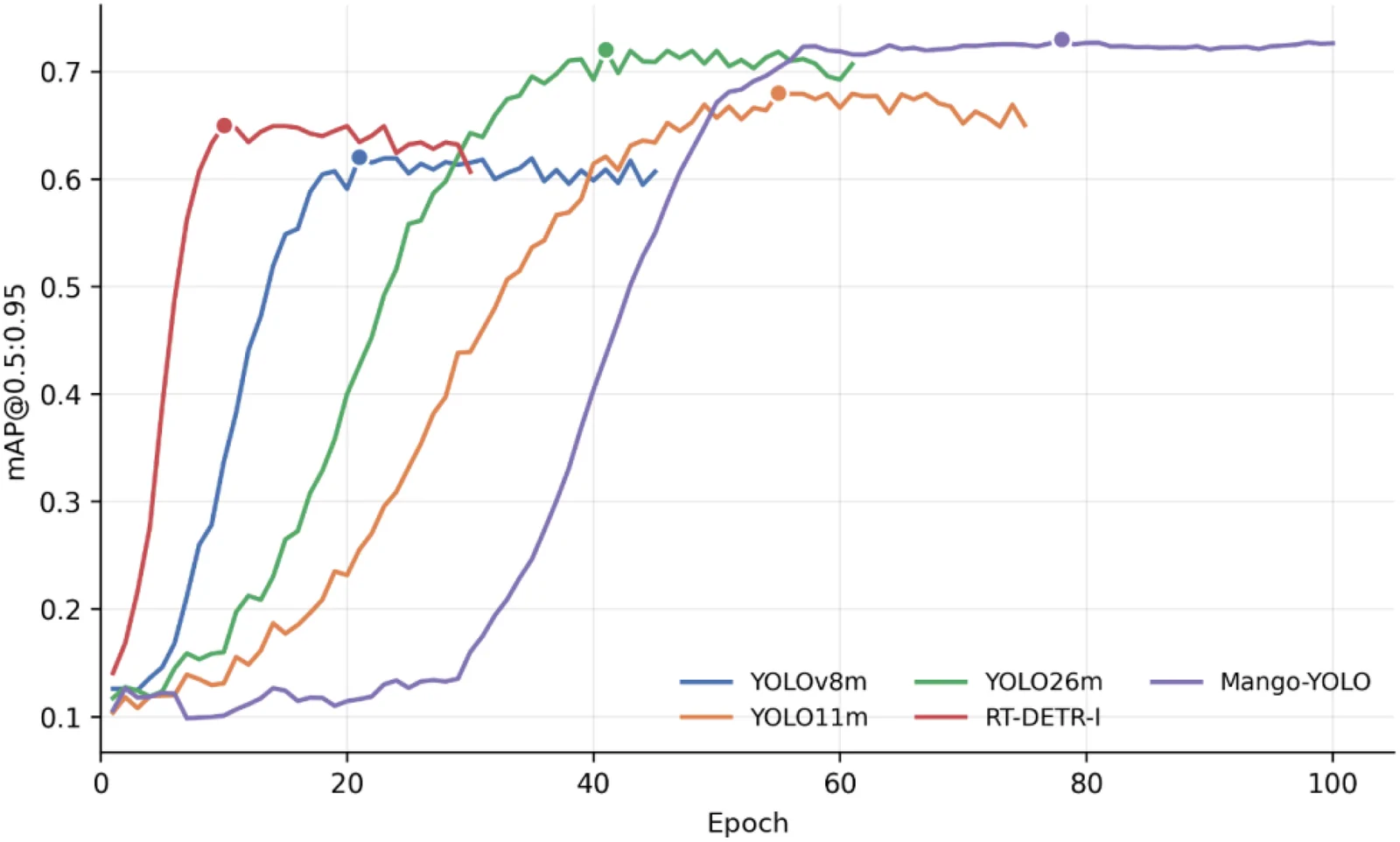
Evolution of mAP@0.5:0.95 on the validation set during training. The marker indicates the best epoch of each model.

The curves reveal differentiated behaviour between architectures. RT-DETR-l shows a very rapid rise in the first epochs, consistent with its nature as a pretrained Transformer-based detector, but stabilizes at a level below that of YOLO26m. The latter presents a more gradual and sustained rise, reaching its optimum at epoch 41. YOLO11m requires the largest number of epochs among the baselines to reach its maximum, at epoch 55. In the four baseline models the difference between the last epoch and the best epoch is constant and equal to 20, except in YOLOv8m where it rises to 24, which confirms that training halted through the early stopping mechanism after exhausting the configured patience.

The behaviour of Mango-YOLO is qualitatively distinct. Its curve presents the slowest start of the set: during the first twenty-five epochs it remains at values close to 0.10 mAP@0.5:0.95, well below the models initialized with COCO pretrained weights, reflecting that the network must learn low-level representations from scratch. From epoch 30 onwards it begins a sustained and pronounced rise that places it above all reference architectures by around epoch 60, and it reaches its optimum at epoch 78 with a mAP@0.5:0.95 of 0.730. During the remaining 22 epochs the curve describes a stable plateau, without further improvement of the validation metric, which evidences that the model attained effective convergence within the assigned budget. It was therefore the latest-converging model of the set, with a best epoch 7.8 times higher than that of RT-DETR-l and 1.9 times higher than that of YOLO26m.

Analysis of the loss functions rules out overfitting as an alternative explanation of the results. In the evaluated models, the training and validation curves for box regression loss, classification loss and distribution focal loss descend jointly and maintain a reduced and stable separation throughout the process. The absence of a growing divergence between the two curves, that is, of a sustained decrease in training loss accompanied by a rise in validation loss, indicates that the models did not incur overfitting within the number of epochs executed, and confirms that the combination of dropout and data augmentation applied was adequate.

### 3.3 Localisation gap

**Figure 9** contrasts, for each model, the value of mAP@0.5 with that of mAP@0.5:0.95, making explicit the magnitude of the gap between the two metrics.

**Figure 9:**
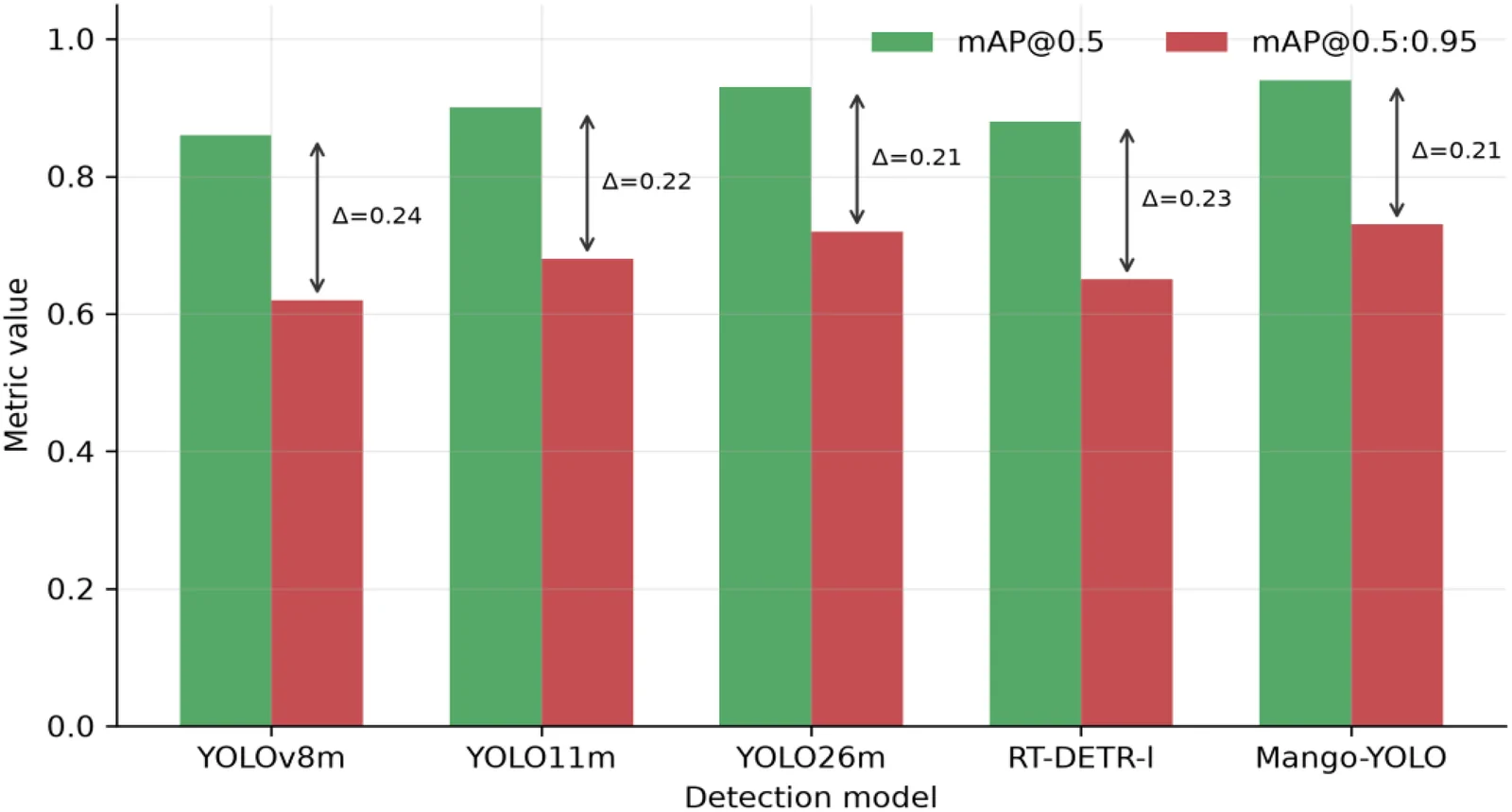
Gap between mAP@0.5 and mAP@0.5:0.95 for each evaluated model.

The gap ranges between 0.210 points, a value shared by Mango-YOLO and YOLO26m, and 0.240 points in YOLOv8m, and occurs systematically across the five architectures. Its interpretation is relevant: the models correctly identify and classify the fruit under a permissive overlap threshold, but the fine adjustment of the bounding box coordinates is considerably less precise. An inverse association is further observed between overall performance and the magnitude of the gap: the two models with the highest F1-score are also those recording the smallest gap, which suggests that localisation quality constitutes a differentiating factor between architectures and not a uniform error attributable exclusively to the dataset.

### 3.4 Per-category performance

The global metrics presented so far average performance over the three categories and, given the documented imbalance, are dominated by the sea freight class. To examine the behaviour of the model in each category separately, the confusion matrix of Mango-YOLO was constructed on the test set, using a confidence threshold of 0.25 and an intersection over union threshold of 0.50. The confidence threshold of 0.25 was used as the conventional Ultralytics operating criterion for retaining detections when constructing the matrix; it was not selected through a separate precision-recall calibration. The IoU threshold of 0.50 corresponds to the standard mAP@0.5 criterion and was used to match each prediction to a ground-truth box of the same class. The matrix incorporates a fourth row and a fourth column termed background, necessary because in a detection problem, unlike a pure classification problem, there exist two types of error that a three-by-three matrix cannot represent: the background column collects false positives, that is, detections emitted by the model in regions where no annotated fruit existed, and the background row collects false negatives, that is, fruit present in the image that the model failed to detect. The matrix is normalized by column, as shown in Equation 27:

(27) $$C_{ij}=n_{ij}/\sum_{i}n_{ij}$$

where the indices i and j range over the categories air freight, sea freight, reject and background. The elements of the main diagonal correspond to correct predictions, and the off-diagonal elements to confusions between categories. **Figure 10** presents the normalized confusion matrix.

**Figure 10:**
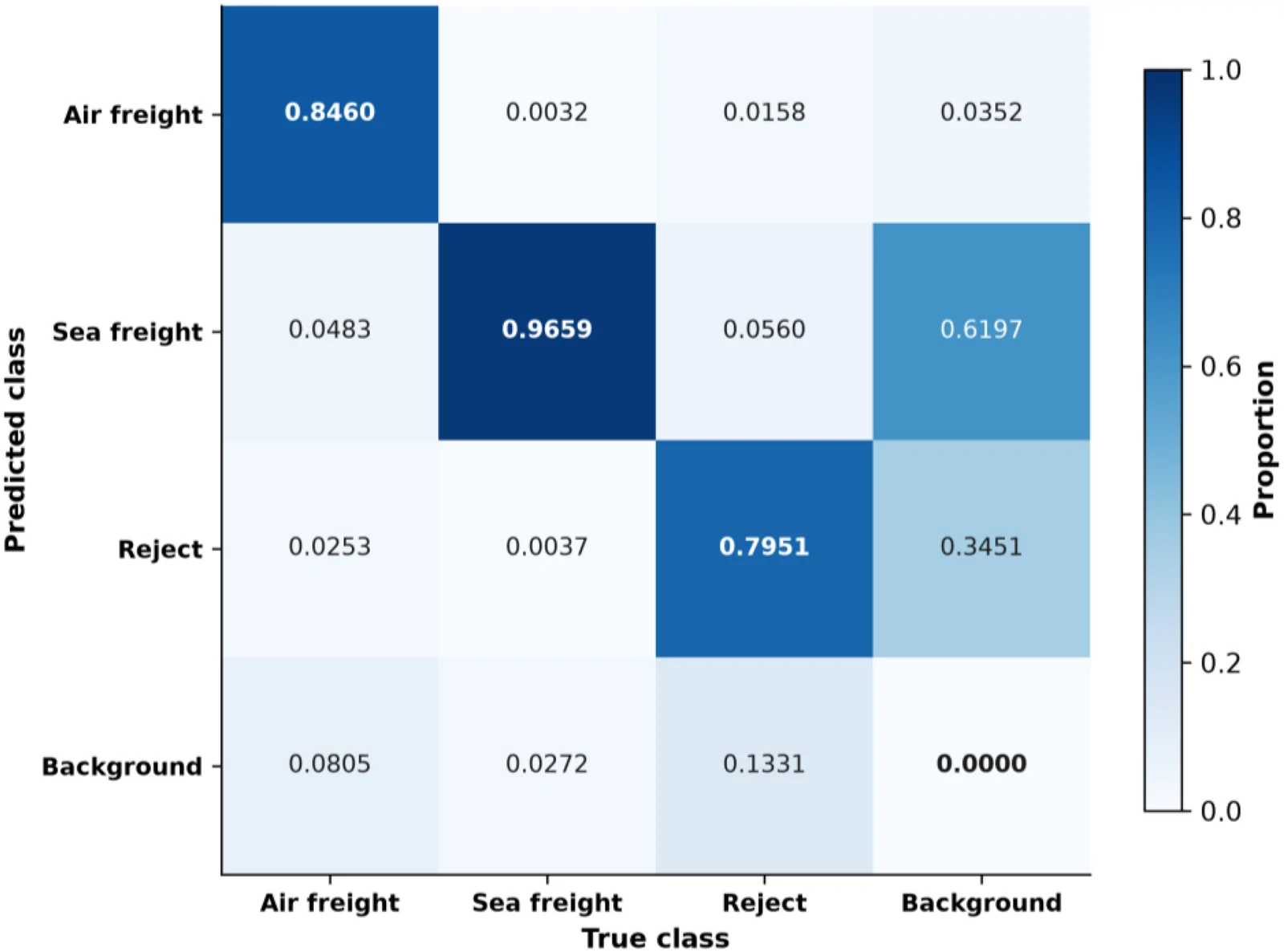
Normalised confusion matrix of the Mango-YOLO model on the test set (confidence threshold 0.25, IoU threshold 0.50).

**Table 8** details the metrics derived from the confusion matrix for each category. The evaluation reveals that model performance is not homogeneous across classes: the F1-score reached 0.960 in the sea freight category, 0.845 in air freight and 0.792 in reject. Since the sea freight category concentrates 86.1% of the instances, the global metrics are dominated by its performance and should not be interpreted as a guarantee of equivalent behaviour in the minority categories, particularly in air freight, which corresponds to the fruit of highest commercial value.

**Table 8:** Per-category metrics of the Mango-YOLO model, derived from the confusion matrix.

Category	Actual inst.	TP	FP	FN	Precision	Recall	F1-score
Air freight	435	368	68	67	0.844	0.846	0.845
Sea freight	9,378	9,058	437	320	0.954	0.966	0.960
Reject	1,142	908	242	234	0.790	0.795	0.792
Overall	10,955	10,334	747	621	0.933	0.943	0.938

### 3.5 Processing time

As part of the evaluation of the third specific objective, the inference time of each architecture was measured on 1,280-pixel images, expressed in milliseconds per image and in frames per second (FPS). **Table 9** presents the results. The proposed Mango-YOLO architecture, in addition to achieving the best detection performance, maintains an inference time of 114.94 ms per image (8.7 FPS), very close to that of the fastest model (YOLOv8m, 108.70 ms) and substantially lower than that of its base architecture YOLO26m (178.57 ms). This indicates that the modifications introduced, that is, the CBAM and BiFPN modules and the MPDIoU loss function, improve accuracy without appreciably penalizing processing speed, which is favourable for deployment in the web application.

**Table 9:** Inference time of the evaluated models on 1,280-pixel images.

Model	Time (ms/image)	Estimated FPS
YOLOv8m	108.70	9.2
Mango-YOLO	114.94	8.7
YOLO11m	123.46	8.1
YOLO26m	178.57	5.6
RT-DETR-l	238.10	4.2

### 3.6 Validation against actual harvested yield

In order to validate the production estimate generated by the system, the automated estimates of the selected model were compared against the actual production harvested and recorded on the processing plant scale for the ten evaluated farms. Actual production per farm was obtained as the sum of the air freight and sea freight categories effectively harvested, while the model estimate corresponds to the projection generated by the Mango Smart system from the automatic fruit count scaled to the total number of plants on the farm. **Table 10** presents both values and the associated percentage error.

**Table 10:** Comparison between actual harvested production and model estimate by farm.

Farm	Plants	Actual harvested (kg)	Model estimate (kg)	Error (%)
F01	7,500	86,189.2	86,411.7	+0.3
F02	2,500	114,624.3	116,873.4	+2.0
F03	500	28,339.7	28,753.1	+1.5
F04	1,200	27,721.8	27,957.1	+0.8
F05	500	20,221.1	21,006.5	+3.9
F06	600	10,550.3	10,184.9	−3.5
F07	550	33,323.0	34,738.9	+4.2
F08	130	3,401.5	3,352.1	−1.5
F09	450	11,774.3	12,545.4	+6.5
F10	400	9,504.2	9,708.4	+2.1
Total	14,330	345,649.4	351,531.6	+1.7

Four standard regression metrics were used to quantify the accuracy of the estimate. The mean absolute error (MAE) measures the average magnitude of the error in the units of the variable, as shown in Equation 28:

(28) $$MAE=\left(1/n\right)\cdot \sum \left|y_{i}-{\hat{y}}_{i}\right|$$

The root mean squared error (RMSE) penalizes large errors more severely, as shown in Equation 29:

(29) $$RMSE=\surd \left[\left(1/n\right)\cdot \sum \;{\left(y_{i}-{\hat{y}}_{i}\right)}^{2}\right]$$

The mean absolute percentage error (MAPE) expresses the error in relative terms, as shown in Equation 30:

(30) $$MAPE=\left(100/n\right)\cdot \sum \left|y_{i}-{\hat{y}}_{i}\right|/y_{i}$$

The coefficient of determination (R^2^) measures the proportion of the variance of actual production explained by the estimate, as shown in Equation 31:

(31) $$R^{2}=1-\left[\sum {\left(y_{i}-{\hat{y}}_{i}\right)}^{2}/\sum {\left(y_{i}-\bar{y}\right)}^{2}\right]$$

where y_i is the actual production of farm i, ${\hat{y}}_{i}$ the model estimate, $\bar{y}$ the mean of actual production and n = 10 the number of farms. The values obtained are presented in **Table 11**. The model reached a coefficient of determination of 0.9993 and a mean absolute percentage error of 2.63%, indicating an almost perfect correspondence between the automated estimates and the actual harvested production. The overall difference between the estimated total (351,531.6 kg) and the actual total (345,649.4 kg) was only 1.7%, with per-farm errors ranging between −3.5% and +6.5%.

**Table 11:** Validation metrics of the yield estimate against actual production.

Metric	Value	Interpretation
MAE	671.2 kg	Mean error per farm in absolute units.
RMSE	934.2 kg	Root mean squared error, sensitive to large deviations.
MAPE	2.63%	Mean relative error with respect to actual production.
R^2^	0.9993	Proportion of the actual variance explained by the model.

**Figure 11** shows the dispersion between both values, in which the ten farms lie practically on the ideal identity line, and **Figure 12** presents the estimation error by farm. The fact that this correspondence is maintained over a production range spanning from 3,401 to 114,624 kilograms indicates that the scaling factor from the sample to the total number of plants operates stably regardless of the size of the productive unit.

**Figure 11:**
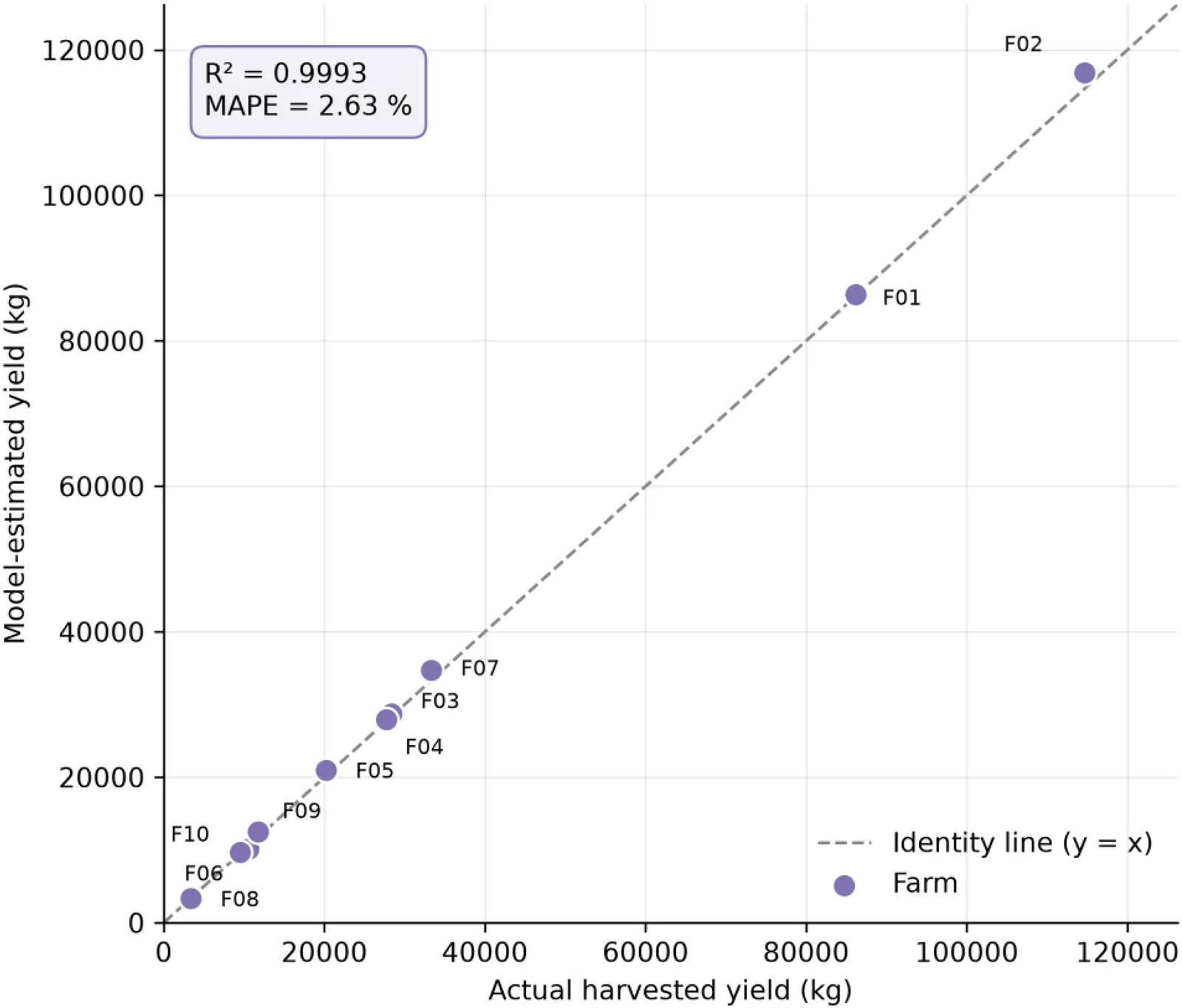
Dispersion between actual harvested production and model-estimated production for the ten evaluated farms.

**Figure 12:**
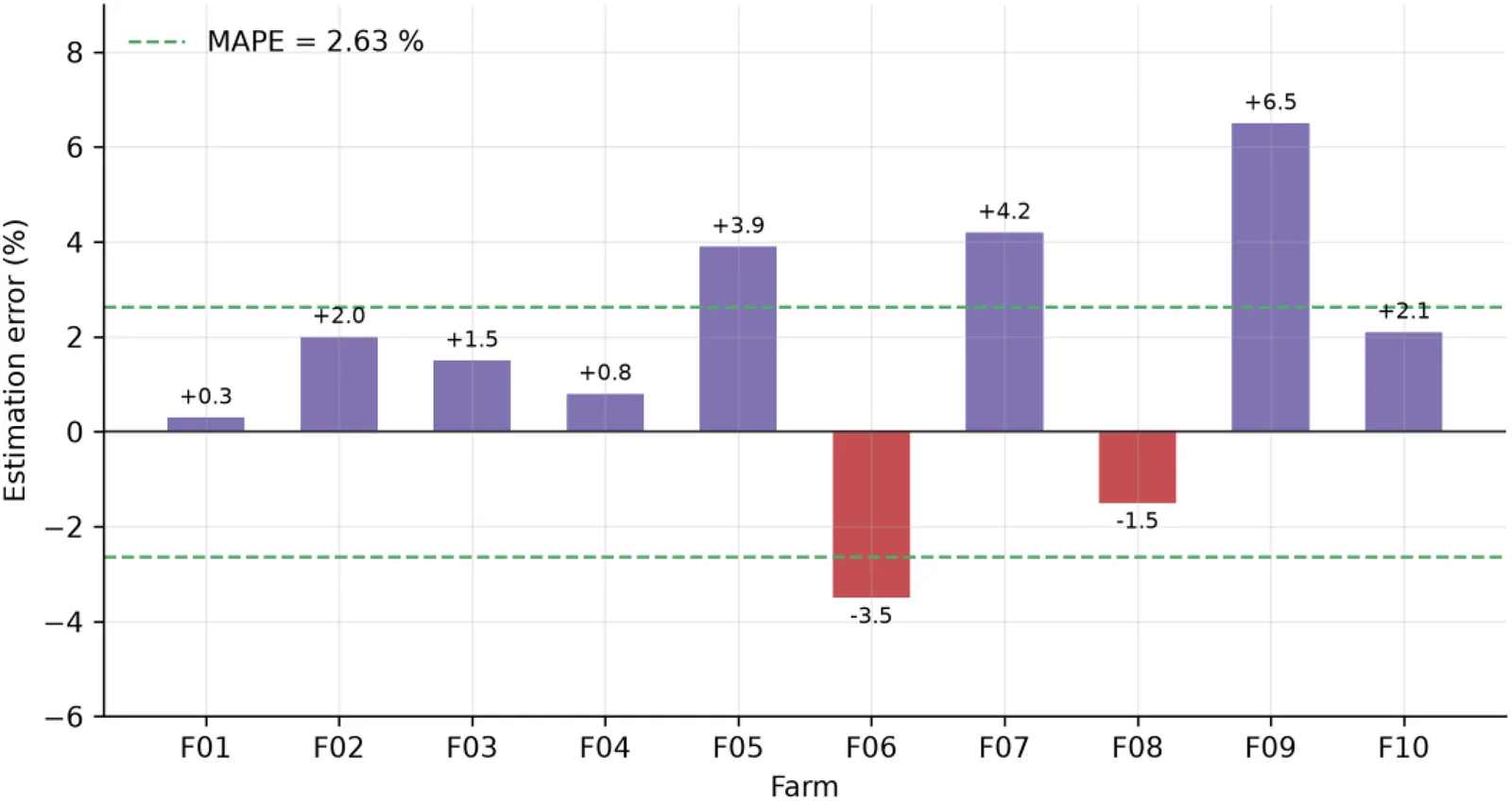
Estimation error of the model by farm, relative to the mean absolute percentage error of 2.63%.

Additionally, the performance of the model was compared against the manual estimate made by the field supervisor, with the aim of contrasting the proposed automated method against the traditional procedure. **Table 12** presents the metrics of both approaches for the sea freight category, which concentrates the greater part of production.

**Table 12:** Performance of the model and of the field supervisor relative to actual production (sea freight category).

Method	MAE (kg)	RMSE (kg)	MAPE (%)	R^2^
Mango-YOLO model	831.8	1,217.5	2.73	0.9987
Field supervisor	29,080.0	84,403.0	88.03	negative
Supervisor (excluding F07)	2,680.0	3,691.0	8.90	0.9896

The results evidence that the automated method substantially outperforms the manual estimate. The manual procedure recorded a mean absolute percentage error of 88.03% and a negative coefficient of determination, a result attributable to an extreme error committed on farm F07. Even excluding that observation, a scenario deliberately favourable to the traditional method, the model maintains a mean absolute percentage error (MAPE) of 2.73%, more than three times lower than that of the human supervisor (8.90%). **Figure 13** illustrates this difference by farm.

**Figure 13:**
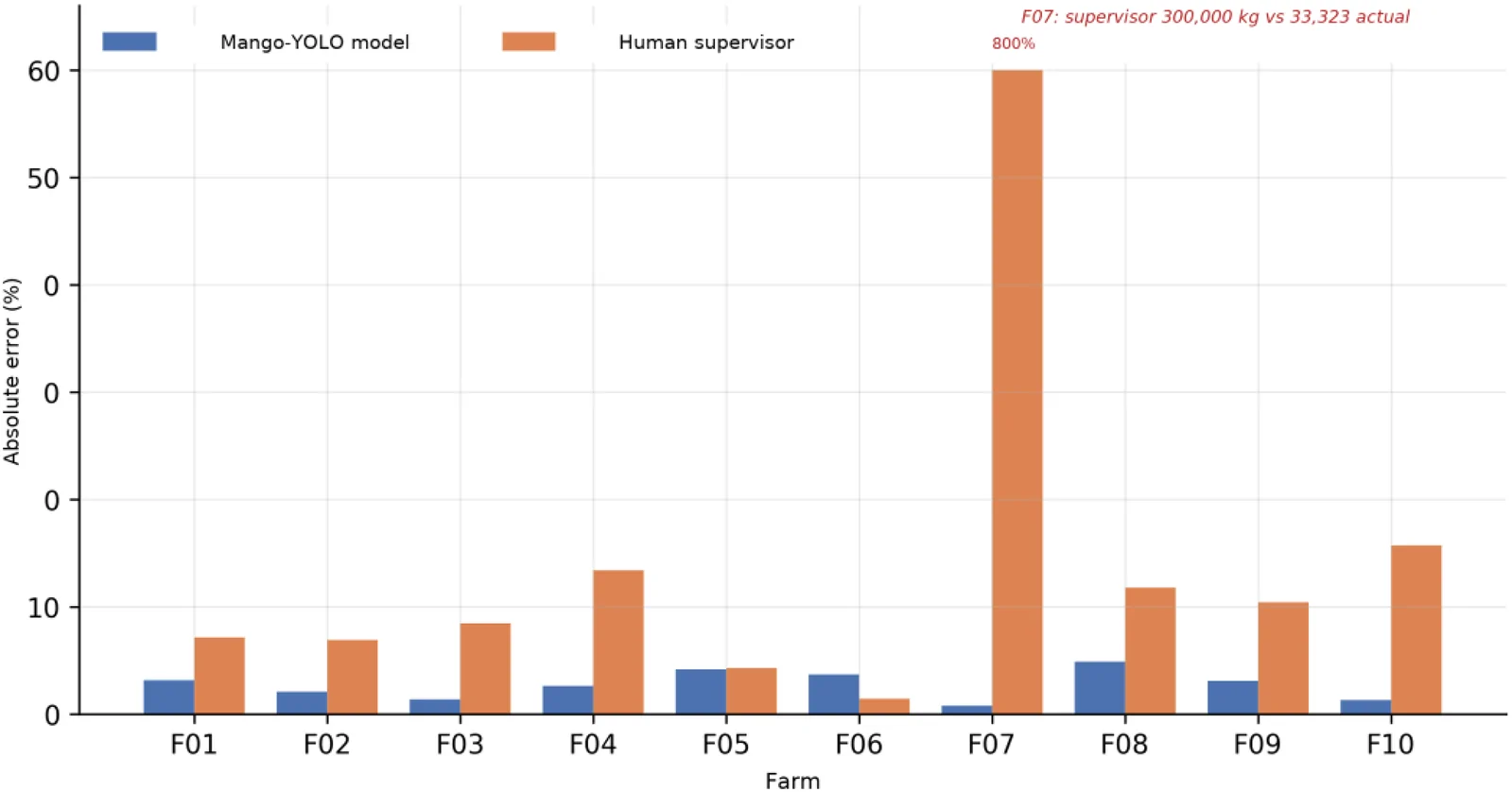
Absolute percentage error of the Mango-YOLO model and of the field supervisor relative to actual production, by farm. The extreme value recorded by the supervisor on farm F07 (approximately 800%, truncated for scale) corresponds to a single manual estimate of 300,000 kg against an actual harvest of 33,323 kg; even excluding this outlier, the model outperforms the supervisor on every remaining farm.

## 4 Discussion

The results obtained confirm the hypothesis put forward. The Mango-YOLO model reached an F1-score of 0.930 and a mAP@0.5 of 0.940 in the detection and classification of Kent mango fruit into three commercial categories, which supports the technical feasibility of replacing manual visual counting with an automated procedure under the field conditions of the Motupe district.

The precision value of 0.930 obtained by Mango-YOLO lies below the 96.72% reported for YOLOv8 in mango detection (Arcila-Díaz et al., 2024) and the 98.20% achieved with Faster R-CNN and ResNet-50 (Vasanth and Pandian, 2024). This comparison must nevertheless be qualified by a substantive difference in the definition of the problem: the cited works address binary detection of mango against background, whereas the present research simultaneously resolves detection and classification into three commercial categories whose visual differentiation is subtle, particularly between air freight and sea freight. The recall of 0.940 obtained considerably exceeds the 77.4% reported for YOLOv8 and the 63.80% of Faster R-CNN in the same comparative study (Arcila-Díaz et al., 2024), which is especially relevant in this application, since an undetected fruit translates directly into an underestimation of production. The recorded performance also exceeds the 85.7% precision obtained in the maturity recognition and counting of sweet peppers (Viveros Escamilla et al., 2024), which constitutes the closest antecedent in terms of the category classification task, and approaches the 94.96% achieved in olive detection in Tacna (Osco-Mamani et al., 2025), the most comparable Peruvian work under field conditions.

The analysis of the training curves contributes an additional element to the debate on architecture selection. RT-DETR-l reached its optimum at epoch 10, 7.8 times faster than Mango-YOLO, which confirms the data efficiency that the literature attributes to Transformer-based detectors (Zhao et al., 2024). That accelerated convergence nevertheless stabilized at a lower performance level, with an F1-score of 0.875 against 0.930. In a scenario with computation time constraints, RT-DETR-l would constitute a reasonable alternative; however, since training is performed only once and inference is subsequently executed on the already-trained model, the criterion of final performance prevails over that of convergence speed. Convergence speed and performance ceiling therefore constitute independent dimensions of detector behaviour.

The most relevant result for the discussion is the performance of the proposed architecture, Mango-YOLO, which obtained the best F1-score of the set (0.930), placing it 0.005 points above YOLO26m, the base model on which it was built, and 0.065 points above YOLOv8m, the lowest-performing architecture. The advantage over the base model, modest in absolute magnitude, is nevertheless consistent: it is verified simultaneously across the five evaluated metrics and does not respond to a trade-off between precision and recall. This result confirms the expectation derived from the literature, according to which the incorporation of CBAM (Woo et al., 2018), BiFPN (Tan et al., 2020) and MPDIoU (Ma and Xu, 2023) should improve performance relative to the base architecture. It must be acknowledged, however, that a difference of 0.005 F1-score points is insufficient, on its own, to attribute a conclusive superiority to the proposed architecture in the absence of an ablation study that isolates the contribution of each component.

This interpretation is coherent with two conditions of the experimental design. First, Mango-YOLO was trained from a configuration defined by a YAML file, without initialization from COCO pretrained weights, whereas the four reference architectures did apply transfer learning (Pan and Yang, 2010). The absence of this initialization forces the network to learn low-level representations from scratch, which substantially increases the number of epochs required to converge. Second, the data augmentation configuration applied to Mango-YOLO was deliberately more aggressive than that of the baselines, which increases the apparent variability of the training set and further slows convergence. The combination of both factors explains why the proposed architecture required a substantially larger number of epochs than the baselines, and accounts for its optimum being reached at epoch 78 against the 41 of YOLO26m, without this preventing its effective convergence within the 100-epoch budget assigned equally to all experiments.

Regarding localisation quality, the systematic gap of between 0.210 and 0.240 points between mAP@0.5 and mAP@0.5:0.95 observed across all models constitutes the most consistent technical limitation of the results. This behaviour is characteristic of the detection of small and partially occluded objects, and is precisely the phenomenon that the MPDIoU loss function incorporated in Mango-YOLO contributes to mitigating (Ma and Xu, 2023). The fact that Mango-YOLO combines the best overall performance with the smallest localisation gap of the set, a value it shares with its base architecture YOLO26m, supports the effectiveness of the modifications introduced.

The class imbalance constitutes an additional factor conditioning the interpretation of the results. The air freight category, which represents the fruit of highest commercial value, concentrates only 3.7% of the annotated instances, against 86.1% for the sea freight category. The majority class exceeds the minority class by a factor of 23.1, which implies that the model has substantially fewer examples available to learn the visual characteristics of the commercially most relevant category. The reported global metrics, being averaged over the three classes, are dominated by performance in the sea freight category and should therefore not be interpreted as a guarantee of equivalent performance in the detection of air freight category fruit. To this condition is added the variability between farms: the proportion of air freight fruit ranges between 14.0% on farm F01 and 0.2% on F07. This means that the model learns the characteristics of this category from a reduced number of cultivation fields, which compromises its generalization capacity to farms with agronomic management conditions different from those represented in the training set. This behaviour is consistent with what was observed in the deployed application, where the estimate performed on a test farm recorded no detections of the air freight category.

Finally, it is necessary to delimit the scope of the results presented. The evaluation carried out measures the capacity to detect and classify the fruit visible in the photographs, not the actual production of the tree. The literature has consistently documented that computer vision methods underestimate fruit load owing to fruit hidden by foliage, with errors reaching 26% when no correction is applied (Anderson et al., 2021), and that correcting the number of detections is a necessary step in yield estimation from images (Sarron et al., 2023). In the present research, the capture of two opposite views per tree partially mitigates this effect, but does not eliminate it. Consequently, the detection metrics reported should not be interpreted as a direct measure of the accuracy of the production estimate in kilograms, even though the validation against the weights recorded by the processing plant yielded a mean absolute percentage error of 2.63%. The convergence of both results suggests that, under the conditions evaluated, the underestimation attributable to occlusion is partially compensated by the scaling factor from the sample to the total number of plants, a compensation that has not been isolated experimentally and that constitutes a priority line for future work.

## 5 Conclusion

This research demonstrates that it is possible to optimize the estimation of Kent mango production by commercial category through the combination of machine learning techniques and image analysis under the real field conditions of the Motupe district. The contribution of the work is articulated on three levels. Methodologically, a dataset of Kent mango captured under Peruvian field conditions with annotation by commercial export category was built and documented, comprising 66,465 instances, a resource that addresses a gap identified in the review of antecedents. Architecturally, Mango-YOLO was proposed and validated, an adaptation of YOLO26m through CBAM attention, BiFPN multi-scale fusion and MPDIoU loss, which outperformed four state-of-the-art reference architectures across all evaluated metrics without an increase in inference cost. In applied terms, Mango Smart was implemented, a three-tier web system that translates the automatic fruit count over a sample of plants into a production estimate expressed in kilograms through Monte Carlo simulation with category mean weights and accompanied by a 90% interval.

The principal quantitative achievements are an F1-score of 0.930 and a mAP@0.5 of 0.940 in the joint task of detection and classification into three commercial categories, and a mean absolute percentage error of 2.63% with a coefficient of determination of 0.9993 in the production estimate against the weights recorded on the scale, a performance 3.3 times more accurate than that of the field supervisor in the scenario most favourable to the latter.

Current limitations delimit the scope of these results with precision. Performance on the air freight category, commercially the most valuable, rests on only 3.7% of the annotated instances and on a reduced number of farms, which compromises its generalization. The evaluation quantifies the detection of visible fruit and not the actual fruit load of the tree, so that underestimation from foliar occlusion remains partially mitigated by two-view capture, but unresolved. The absence of an ablation study prevents the observed improvement from being attributed to each of the three incorporated components separately. Finally, the evidence derives from a single district and a single agricultural season.

Future work should prioritize: widening image capture on farms with agronomic management oriented towards optimal-calibre fruit in order to raise the representation of the air freight category; incorporating an occlusion correction factor calibrated against destructive counts of whole trees; executing an ablation study that isolates the marginal contribution of CBAM, BiFPN and MPDIoU; evaluating input resolutions above 640 × 640 pixels to reduce the localisation gap; and extending validation to farms in other producing districts and to successive agricultural seasons, increasing the number of productive units with actual production recorded on the scale beyond the ten paired observations of the present study.

## Data Availability

The raw data supporting the conclusions of this article will be made available by the authors, without undue reservation.
